# Bildgebung des postoperativen Beckenbodens

**DOI:** 10.1007/s00117-023-01203-x

**Published:** 2023-09-12

**Authors:** Antonia M. Pausch, Cornelia Betschart, Andreas M. Hötker

**Affiliations:** 1 Institut für Diagnostische und Interventionelle Radiologie, Universitätsspital Zürich, Rämistr. 100, 8091 Zürich, Schweiz; 2 Klinik für Gynäkologie, Universitätsspital Zürich, Zürich, Schweiz

**Keywords:** Genitaldeszensus, Deszensuschirurgie, Mesh, Magnetresonanztomographie, Computertomographie, Pelvic organ prolapse, Prolapse surgery, Mesh, Magnetic resonance imaging, Computed tomography

## Abstract

**Klinisches/methodisches Problem:**

Der Genitaldeszensus ist ein häufiges Krankheitsbild der Frau, wobei zur Therapie sowohl konservative als auch chirurgische Maßnahmen zur Verfügung stehen. Für die adäquate radiologische Diagnostik nach stattgehabter Deszensuschirurgie ist die Kenntnis der verschiedenen Operationsverfahren und des eingesetzten Fremdmaterials essenziell, um mögliche Komplikationen von normalen postoperativen Veränderungen zu unterscheiden.

**Radiologische Standardverfahren:**

Im unmittelbaren postoperativen Verlauf ist die Computertomographie (CT) zur Evaluation akuter Komplikationen wie Blutungen oder Organverletzungen meist die gewählte Modalität. Die Magnetresonanztomographie (MRT) bietet einen hohen Weichteilkontrast und ist daher in der Regel zur Beurteilung subakuter und chronischer Komplikationen zu bevorzugen.

**Methodische Innovationen:**

Innovative Techniken wie dynamische MRT-Protokolle können die radiologische Beurteilung nach Deszensuschirurgie verbessern und beispielsweise die Bewertung der Organmobilität unter Belastung ermöglichen.

**Leistungsfähigkeit:**

Radiologische Standardverfahren wie CT und MRT liefern detaillierte Informationen über den postoperativen Situs und potenzielle Komplikationen nach Deszensuschirurgie.

**Bewertung:**

Die radiologische Bildgebung spielt insbesondere bei Komplikationen eine wichtige Rolle bei der Evaluation von Patientinnen nach Deszensuschirurgie. Durch eine präzise radiologische Diagnosestellung können adäquate weitere Therapiemaßnahmen ergriffen werden.

## Abstract (en)

**Clinical/methodical issue:**

Pelvic organ prolapse is a common condition in women, for which both conservative and surgical interventions are available. Knowledge of the different surgical procedures and the materials used is essential for adequate radiological diagnosis after prolapse surgery in order to differentiate potential complications from normal postoperative changes.

**Standard radiological methods:**

In the immediate postoperative period, computed tomography (CT) is often the modality of choice for evaluating acute complications such as bleeding or organ injuries. Magnetic resonance imaging (MRI) provides excellent soft tissue contrast and is therefore generally preferred for assessing subacute and chronic complications.

**Methodical innovations:**

Innovative techniques such as dynamic MRI protocols can improve the radiological assessment after prolapse surgery by enabling the evaluation of organ mobility.

**Performance:**

Radiological standard procedures such as computed tomography (CT) and MRI provide detailed and reliable information about the postoperative site and potential complications following prolapse surgery.

**Achievements:**

Radiological imaging plays an important role in the evaluation of patients after prolapse surgery, particularly when complications are suspected. Accurate radiological diagnosis can guide further appropriate therapeutic measures.

Der Genitaldeszensus ist ein häufiges Krankheitsbild der Frau mit einer Prävalenz von ca. 30–50 %, wobei die exakten Fallzahlen abhängig von der genauen Definition, den Risikofaktoren einer Frau und dem Schweregrad des Deszensus variieren [1]. Zugrundeliegend ist eine Schwäche der muskulären und ligamentären Strukturen des Beckenbodens [2], welcher in das anteriore, mittlere bzw. apikale und posteriore Kompartiment eingeteilt wird. Der Descensus genitalis kann sich entweder insoliert in einem dieser Kompartimente, aber auch unter Beteiligung mehrerer Kompartimente manifestieren: Hierbei kann ein Absinken der Blase (Zystozele) im vorderen Kompartiment, der Vagina oder des Uterus im mittleren bzw. apikalen Kompartiment und des Rektums (Rektozele) im hinteren Kompartiment beobachtet werden. Wenn die Absenkung der genannten Organe über den Introitus hinausgeht, wird dies im deutschsprachigen Raum als Prolaps bezeichnet [3].

## Diagnose und Therapiemöglichkeiten

Die Häufigkeit des Genitaldeszensus hängt von verschiedenen Faktoren ab, darunter beispielsweise das Alter, die Anzahl der vorangegangenen vaginalen Geburten und das Gewicht einer Frau [4], wobei geschätzt wird, dass Frauen im Laufe ihres Lebens ein Risiko von ca. 20 % haben, bis zum Alter von 80 Jahren eine chirurgische Deszensuskorrektur zu benötigen [5]. Typische, von den Patientinnen geschilderte Symptome sind Blasen- oder Darmentleerungsstörungen, ein störendes Senkungsgefühl mit Körperbildveränderungen, sexuelle Dysfunktion und Schmerzen, wobei – abhängig vom Schweregrad der Erkrankung – die Lebensqualität hierdurch teils deutlich eingeschränkt sein kann [6].

Im deutschsprachigen Raum bietet die S2e-Leitlinie „Weiblicher Descensus genitalis, Diagnostik und Therapie“ [3] einen umfangreichen Überblick über diagnostische Maßnahmen und Therapieoptionen und stellt wichtige Empfehlungen für die adäquate und individualisierte Versorgung der Patientinnen bereit. Um gemeinsam eine sinnvolle Entscheidung über die Behandlung zu treffen, sollten daher neben klinischen Befunden auch die Bedürfnisse und Präferenzen der Patientinnen berücksichtigt werden.

Als konservative Therapiemaßnahmen stehen neben Programmen zur Beckenbodenrehabilitation und dem Einsatz von Pessaren auch ein abwartendes Vorgehen bzw. die klinische Beobachtung und die Reduktion von eventuellen Risikofaktoren zur Verfügung. Chirurgische Therapieansätze sollten im Allgemeinen erst angestrebt werden, wenn konservative Therapieansätze scheitern und ein entsprechender Leidensdruck bzw. Symptome bestehen [3].

## Operationstechniken

Die chirurgische Therapie des Genitaldeszensus hat sich im Laufe der Zeit stetig weiterentwickelt. Mittlerweile stehen mehrere operative Techniken mit der Verwendung von Eigengewebe und Implantaten sowie unterschiedliche Zugangswege zur Verfügung.

### Operationstechniken mit autologem Gewebe

Die Operationen mit Eigengewebe bieten sich im vaginalen Zugang an und sind seit vielen Jahrzehnten verbreitet. Kompartimentspezifisch wird im Fall einer Zystozele die vesikopelvine Faszie, auch endopelvine Faszie genannt, vom Apex der Vagina bis zum Blasenhals dupliziert, womit der zentrale Defekt der Zystozele verschlossen wird (Diaphragmaplastik). Das Pendant im posterioren Kompartiment ist die Kolpoperineoplastik und beruht auf der Verstärkung der Fascia rectovaginalis zur Behebung der Rektozele. Werden zusätzliche anatomische Landmarks zur apikalen Fixierung, wie z. B. das Ligamentum sacrospinale oder die Ligamenta sacrouterina miteinbezogen, wird auch das apikale Kompartiment fixiert und der Outcome der Operation verbessert sich, so dass meistens Kombinationsoperationen erfolgen. Die autologen Operationen werden grundsätzlich mit resorbierbaren Fäden durchgeführt und führen zu einer lokalen Fibrose [2, 3, 7].

### Meshbasierte Deszensuskorrekturen

Aufgrund des dem Eigengewebe inhärenten Rezidivrisikos haben sich in den vergangen zwei Dekaden meshbasierte Deszensusoperationen etabliert, welche heutzutage zur Vermeidung von vaginalen Mesherosionen primär laparoskopisch oder roboterunterstützt durchgeführt werden. Das am meisten verwendete Material ist Polypropylen, das in vorgefertigten Kits mit oder ohne Titanisierung erhältlich ist.

Bei der Sakrokolpopexie wird die anteriore und posteriore Vaginalwand mit einem Y‑förmig verlaufenden Mesh unterlegt und fixiert. Das Mesh zieht die Vagina nach kranial an das anteriore Lig. longitudinale am Sakrum (Promontorium oder SWK 1–3). Initial wurde nur der Apex fixiert, mittlerweile hat sich eine zusätzlich anteriore und posteriore Fixierung bis zum Blasenhals bzw. posterior tief hinunter bis zum Levator ani beidseits durchgesetzt, was das Risiko für ein kaudales Zysto- oder Rektozelenrezidiv verringert, aber Blasen‑, Darm- oder Ureterläsionen im Falle von postoperativen Beschwerden wahrscheinlicher werden lässt.

## Bildgebende Verfahren

Zur adäquaten Beurteilung der Bildgebung nach stattgehabter Beckenbodenchirurgie ist die Kenntnis der verschiedenen Operationsverfahren und des eingesetzten Fremdmaterials essenziell, um mögliche Komplikationen von normalen postoperativen Veränderungen zu unterscheiden. Beim Verdacht auf intraoperative bzw. unmittelbare postoperative Komplikationen kann der/die Operateur/-in dem/-r Radiolog/-in allenfalls detaillierte Informationen über den Operationsverlauf zur Verfügung stellen. Bei extern zugewiesenen Patientinnen mit möglicherweise bereits länger zurückliegender Deszensuschirurgie können diese Angaben jedoch auch fehlen und somit die konklusive Interpretation der Bildgebung nochmals erschweren. Unter Berücksichtigung der vorliegenden Informationen ist die Wahl der geeigneten Bildgebungsmodalität sowohl von der Art der vermuteten Komplikation als auch dem Zeitpunkt des erstmaligen Auftretens abhängig [8].

### Computertomographie

Im unmittelbaren postoperativen Verlauf ist die Computertomographie zur weiteren Abklärung akuter Komplikationen wie Blutungen, Organverletzungen oder einer Darmpassagestörung meist die Modalität der Wahl. Darüber hinaus können jedoch auch subakute oder chronische Komplikationen wie Infektionen und Fistelbildungen beurteilt werden [8, 9]. Abhängig von der genauen Fragestellung werden Einphasen (z. B. postoperative Infektsuche) oder Mehrphasen-CT-Protokolle (z. B. Blutung) gewählt [8, 10].

### Magnetresonanztomographie

Zur Evaluation subakuter oder chronischer Komplikationen nach Deszensuschirurgie ist aufgrund des hohen Weichteilkontrastes im weiblichen Becken die MRT zu bevorzugen [8]. Als Untersuchungsprotokoll empfiehlt sich die Akquisition hochauflösender 2D T2-gewichteter TSE-Sequenzen in 3 Ebenen, 3D T1-gewichteter GRE-Sequenzen nativ und fettgesättigt nach Kontrastmittelapplikation sowie ggf. von diffusionsgewichteten Sequenzen, 3D T2-gewichteten TSE-Sequenzen und suszeptibilitätsgewichteten Sequenzen [8, 11]. Ein vorangehendes Fasten der Patientin ist nicht notwendig, jedoch kann die Gabe eines Spasmolytikums zur Reduktion von Bewegungsartefakten durch Darmmotilität hilfreich sein.

Aufgrund der Komplexität der Beckenbodenfunktionsstörungen kann zur weiteren Evaluation auch die Notwendigkeit einer dynamischen MRT-Untersuchung gegeben sein. Entsprechende Empfehlungen hierfür wurden sowohl von der European Society of Urogenital Radiology und European Society of Gastrointestinal and Abdominal Radiology als auch von der Society of Abdominal Radiology (SAR) formuliert [12, 13]. Statische MR-Bilder veranschaulichen die genaue Anatomie des Beckenbodens und potenzielle Defekte der Haltestrukturen, während dynamische MRT-Bilder zusätzlich die Beweglichkeit der Beckenorgane und eine Beckenbodenschwäche bzw. einen Genitaldeszensus unter Belastung sowie hiermit verbundene Kompartmentdefekte visualisieren [14, 15]. Ausführliche Details hierzu finden sich in einem weiteren Artikel zum Leitthema dieser Ausgabe.

## Normalbefund nach Deszensuschirurgie mit Mesh-Implantation

Wurde bei einer Patientin ein Mesh als Therapieoption gewählt und operativ eingebracht, so kommen die implantierten Netze bildmorphologisch meist als lineare oder tubuläre Strukturen zur Darstellung, die je nach durchgeführter Operation einen unterschiedlichen Verlauf zeigen können [16]: Bei Status nach Sakrokolpopexie ist beispielsweise das zweiarmige Sakrokolpopexie-Netz in Form eines umgekehrten Y mit gewöhnlich nach rechts gekrümmtem Verlauf von den Vaginalwänden bis zum Promontorium zu identifizieren [8], wobei der rechtsgekrümmte Verlauf durch die subperitoneale Positionierung des Meshs bedingt ist. Falls das Netz unter höherer Spannung fixiert wurde, kann es ggf. das Peritoneum leicht pelottieren und einen eher medianen Verlauf durch das Becken zeigen [17]. Bei Status nach TVT-Operation („tension-free vaginal tape“) zur Therapie einer Stressinkontinenz ist das Band – abhängig von der gewählten Operationstechnik – als schlingenartige bzw. U‑förmige periuretrale Struktur mit Verlauf transobturatorisch oder zum retro- und suprapubischen Raum zu identifizieren. Die Enden des Band sind nicht im Knochen verankert, sondern werden spannungsfrei in den Weichteilen fixiert [9].

Die Sichtbarkeit des Meshs in der CT ist abhängig vom verwendeten Material und einer möglichen assoziierten Fibrosierung im Verlauf des Meshs. Daher sind die Mes﻿hs in der CT teils nicht direkt sichtbar und nur durch die assoziierte Narbenbildung identifizierbar, teils jedoch auch als hyperdenses Material direkt abgrenzbar. Insbesondere wenn die eingebrachten Netze sehr dünn sind oder keine entsprechende Anamnese bezüglich einer durchgeführten Beckenbodenchirurgie vorliegt, kann die eindeutige Detektion in der CT möglicherweise nicht gewährleitet sein [8].

In der MRT sind die Meshs meist als lineare bzw. tubuläre T2w-hypointense Strukturen zu identifizieren. Im Mesh-Verlauf lassen sich zudem häufig Suszeptibilitätsartefakte als Korrelat von Hämosiderinablagerungen oder metallischen Clips zur Fixierung finden [17]. Nach Sakrokolpopexie kann beispielsweise die Fixierung des Netzes am Promontorium oft durch eine narbenbedingte Verdickung oder Suszeptibilitätsartefakte identifiziert werden. Eine genaue Differenzierung zwischen Mesh und assoziiertem Narbengewebe, das ebenfalls T2w-hypointens zur Darstellung kommt, ist jedoch nicht immer eindeutig möglich [11].

## Postoperative Komplikationen

Die häufigsten Komplikationen nach Deszensuschirurgie sind vaskulärer Genese im Sinne von Hämatomen oder Blutungen, Infektionen, Verletzungen angrenzender Beckenorgane und Mesh-Dislokationen bzw. Mesh-Defekte [8].

### Blutungen und Hämatombildung

Das Risiko für intraoperative Blutungen bzw. Hämatombildungen wird je nach Operationstechnik und Zugangsweg auf ca. 0,4–4,4 % eingeschätzt [18, 19]. Hierbei sind insbesondere das Promontorium bzw. der präsakrale Raum häufige Blutungslokalisationen, bedingt durch die hier befindliche A. und V. sacralis mediana bzw. den Plexus venosus sacralis [20]. Bei asymptomatischen, Hb-stabilen Patientinnen können im postoperativen Verlauf ggf. auch Mesh-assoziierte Hämatome beobachtet werden; in diesem Kontext ist die Abgrenzung zum Abszess essenziell [16]. Zur Differenzierung ist es daher insbesondere hilfreich zu prüfen, ob abszesstypische Merkmale vorliegen (wie z. B. Gaseinschluss oder randständige Kontrastmittelaufnahme; [21]).

### Mesh-Dislokation bzw. Mesh-Defekt

Im Rahmen einer Mesh-unterstützten Deszensuskorrektur besteht das Risiko einer Mesh-Erosion mit möglicher Extrusion des Netzes [2, 3]. Eine Netzextrusion bezeichnet hierbei eine Wanderung des Netzes durch einen Wanddefekt, bis es in das Lumen eines angrenzenden Organs ragt [22]. Das Risiko einer Mesh-Erosion variiert u. a. abhängig von den individuellen Risikofaktoren der Patientin und wird beispielsweise bei Sakrokolpopexie-Meshs in Studien auf ca. 1–10,5 % bemessen [19, 23]. Während die körperliche Untersuchung bei symptomatischen Patientinnen meist eine sehr große Relevanz in der Beurteilung einer möglichen Netzerosion hat, spielt die Bildgebung insbesondere dann eine wichtige Rolle, wenn das Netz nur teilweise in die Wand eines Organs hineinragt, aber nicht vollständig freigelegt ist [11]. Gegebenenfalls kann die vaginale oder rektale Applikation von Ultraschallgel sinnvoll sein, um durch die Distension das in der MRT T2w-hypointense Mesh vom T2w-hyperintensen Gel besser intraluminal abgrenzen zu können [11]. Eine Ablösung des Meshs von den Fixierungsstellen ist eine eher seltene Komplikation, so kann es jedoch beispielsweise nach Sakrokolpopexie zu einer Mesh-Ablösung vom Promontorium oder der vaginalen Befestigungsstellen kommen [17].

### Infektionen und Fisteln

**Figure 1:**
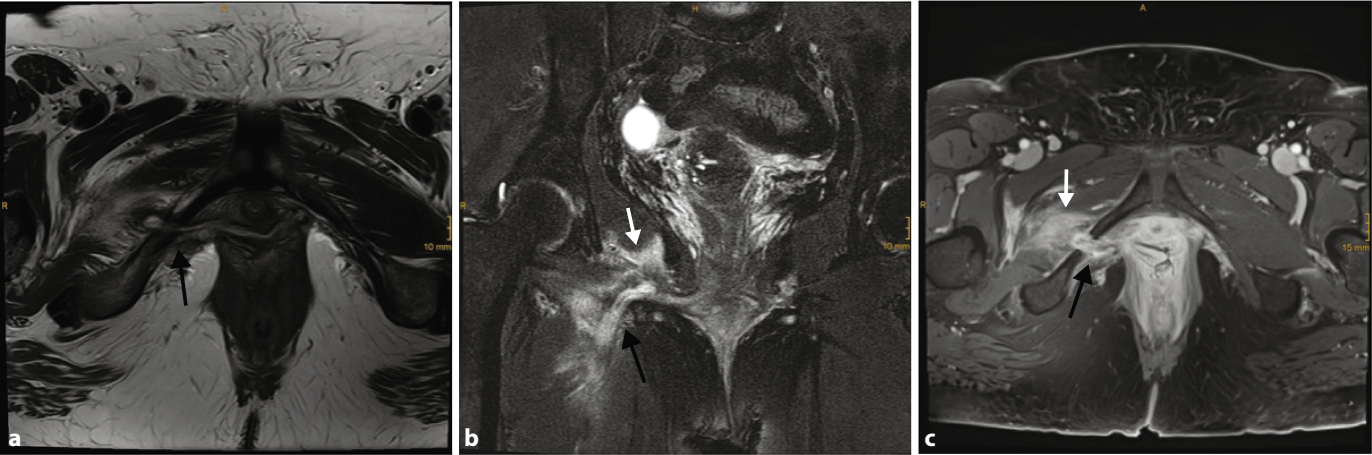

Infektionen sind sowohl eine mögliche Komplikation im frühen als auch erst im späteren postoperativen Verlauf, wobei das Risiko hierfür mit ca. 0,1–0,6 % als gering einzuschätzen ist [18, 23, 24]. Im frühen Verlauf kann es zu Infektionen im Operationsgebiet bzw. operativen Zugangsweg kommen, die möglicherweise auch mit einer Abszessbildung einhergehen. Es ist zu beachten, dass insbesondere Infektionen des Meshs an sich auch erst Jahre nach der Operation auftreten können und dann meist mit einer Mesh-Erosion assoziiert sind [16] – hier ist die Kenntnis der entsprechenden Patientenvorgeschichte für den befundenen Radiologen von größter Bedeutung. MR-tomographisch kann eine Verdickung, flüssigkeitsisointense Signalveränderungen oder vermehrte Kontrastmittelaufnahme des Meshs auf eine Infektion hinweisen [11]. Des Weiteren sollte auch auf mögliche assoziierte Abszessformationen bzw. Fistelbildungen geachtet werden – Letztere können sowohl als Sinustrakt blind enden als auch Anschluss an die Haut gewinnen, bildmorphologisch manifestieren sie sich als gangartige Strukturen mit Wandverdickung, randständiger Kontrastmittelaufnahme, Diffusionsrestriktion in der MRT und möglicherweise Gaseinschlüssen (Abb. 1). Gaseinschlüsse entlang des Meshs könnten ggf. fälschlicherweise als Darmschlinge bewertet werden, daher sollte die Befundbeurteilung stets in mehreren Ebenen erfolgen [8].

Infektionen an der Verankerung des Meshs am Sakrum und Promotorium können in sehr seltenen Fällen mit einer Discitis oder Osteomyelitis einhergehen, nach kombinierter Sakrokolpopexie und Rektopexie wurde diese Komplikation bei 1,7 % der Patienten innerhalb der ersten 6 Wochen nach Operation beobachtet [25]. Bei entsprechendem Verdacht ist zur Diagnosestellung eine dezidierte MRT der Wirbelsäule sinnvoll [26].

### Postoperatives Rezidiv eines Genitaldeszensus

Nach erfolgter Deszensuschirurgie kann es zu einem erneuten Genitaldeszensus im ursprünglich chirurgisch fixierten Kompartiment oder aber auch in einem anderen, zuvor nicht betroffenen Kompartiment kommen [9]. Risikofaktoren für ein postoperatives Rezidiv sind u. a. der präoperative Schweregrad des Deszensus/Prolaps, eine vorhandene Avulsion des M. levator ani und eine positive Familienanamnese [27], des Weiteren kann ein Defekt des eingebrachten Meshs zugrunde liegen [11]. Bei entsprechendem klinischem Verdacht kann ggf. eine dynamische MRT (dMRT) durchgeführt werden. Es konnte gezeigt werden, dass die gemessene Organmobilität im Rahmen der dMRT gut mit den subjektiven Beckenbodenbeschwerden und hiermit assoziierten Einschränkungen der Lebensqualität der Patientinnen korreliert [28].

## Fazit für die Praxis

- Der Genitaldeszensus ist ein häufiges Krankheitsbild unter Frauen mit steigender Tendenz aufgrund der zunehmenden Lebenserwartung.
- Als Therapiemöglichkeiten stehen neben konservativen Ansätzen auch chirurgische Maßnahmen zur Verfügung, wobei teils Meshs zur operativen Rekonstruktion des Beckenbodens verwendet werden.
- Die Kenntnis der angewendeten Operationstechnik ist für die Bildgebung relevant, um postoperative Befunde von Komplikationen zu unterscheiden.
- Im unmittelbaren postoperativen Verlauf wird meist die Computertomographie (CT) zur Abklärung akuter Komplikationen wie etwa Blutungen oder Abszessbildung eingesetzt.
- Mit der Magnetresonanztomographie (MRT) kann hingegen die genaue Anatomie des Beckenbodens und eventuelle Mesh-Defekte bzw. -Dislokationen präzise evaluiert werden.
- Darüber hinaus kann mittels dynamischer MRT auch eine funktionelle Untersuchung zur Beurteilung der Interaktion des Beckenbodens und der Beckenorgane untereinander erfolgen.
